# Superradiant Quantum Phase Transition for an Exactly Solvable Two-Qubit Spin-Boson Model

**DOI:** 10.3390/e25020187

**Published:** 2023-01-17

**Authors:** Roberto Grimaudo, Davide Valenti, Alessandro Sergi, Antonino Messina

**Affiliations:** 1 Dipartimento di Fisica e Chimica “Emilio Segrè”, Group of Interdisciplinary Theoretical Physics, Università degli Studi di Palermo, Viale delle Scienze Ed. 18, 90128 Palermo, Italy; 2 Dipartimento di Scienze Matematiche e Informatiche, Scienze Fisiche e Scienze della Terra, Università degli Studi di Messina, Viale F. Stagno d’Alcontres 31, 98166 Messina, Italy; 3 Institute of Systems Science, Durban University of Technology, P.O. Box 1334, Durban 4000, South Africa; 4 Dipartimento di Matematica ed Informatica, Università degli Studi di Palermo, Via Archirafi 34, 90123 Palermo, Italy

**Keywords:** open quantum systems, two-qubit spin-boson model, exactly solvable models, quantum phase transitions, entanglement, superradiance

## Abstract

A spin-boson-like model with two interacting qubits is analysed. The model turns out to be exactly solvable since it is characterized by the exchange symmetry between the two spins. The explicit expressions of eigenstates and eigenenergies make it possible to analytically unveil the occurrence of first-order quantum phase transitions. The latter are physically relevant since they are characterized by abrupt changes in the two-spin subsystem concurrence, in the net spin magnetization and in the mean photon number.

## 1. Introduction

Every realistic quantum physical system is unavoidably coupled to sources of decoherence and/or dissipation [1]. Open quantum systems are particularly intriguing since their dynamic behaviour provides the platform to study the quantum-classical border.

A basic model exhibiting the quantum dissipation phenomenon is the spin-boson model (SBM), which describes a single spin-1/2 coupled to a quantized bosonic field [2]. The SBM has been deeply investigated through several methods and techniques since the 1980s [2,3,4,5,6,7,8,9,10].

Because of zero-point fluctuations rather than thermal ones [11,12,13], the SBM exhibits quantum phase transitions (QPTs) with respect to both the system-bath coupling and the transverse field strength [4,6,7,8,14,15,16,17,18,19,20]. Despite of its apparent simplicity, the SBM has spurred theoretical and experimental investigations which have successfully explored the basic physics of open quantum systems, significantly contributing to the understanding of different basic aspects that emerge experimentally in the behavior of such systems. Moreover, thanks to its versatility and generality, the SBM is at the basis of numerous applications in several fields, ranging from quantum information, quantum computation, and quantum simulation, to quantum optics and condensed matter physics [21,22,23,24,25].

In the last years a growing attention has been focusing on the decoherent and dissipative dynamics of the two-qubit SBM [17,26,27,28,29]. These models turn out to be useful to describe physical systems consisting of a collection of bi-nuclear units [30,31]. In the two-qubit SBMs until now analysed, either decoupled qubits or the simplest spin–spin coupling have been considered [17,27,32]. The model we are going to investigate in the following sections, instead, presents a non-trivial isotropic Heisenberg interaction between the two spins. The inclusion of such an interaction term is of crucial importance for those systems where the spin–spin interaction cannot be neglected. In particular it plays a crucial role in quantum computation [33,34] and, more generally, for those scenarios where the spin–spin coupling is the key ingredient for both performing controlled gates [35,36] and generating multipartite entangled states [37,38,39].

In this work we show that, thanks to the symmetries exhibited by the Hamiltonian, we are able to find the analytical expressions of both eigenstates and (related) eigenenergies. The dynamical problem can be exactly solved and this fact enables to bring to light the occurrence of first-order QPTs, characterized by abrupt changes of three physical quantities of experimental interest: the level of entanglement between the two spins (estimated through the concurrence [40]), the net two-spin magnetization, and the mean value of the number of bosonic field excitations. In particular, the change from a vanishing to a non-vanishing value of the mean photon number allows to speak of superradiant phase transition. The paper is structured as follows. In Section 2 the general model introduced in Ref. [41] and its symmetry properties are presented. In Section 3 the physical conditions which realize the exchange symmetry between the two spins and which make the model exactly solvable are considered. The analytical expressions of the eigenstates and eigenenergies are further derived, with the occurrence of first-order QPTs with respect to the parameters characterizing the Hamiltonian model. Final comments are reported in the last section.

## 2. Model and Symmetries

**Figure 1:**
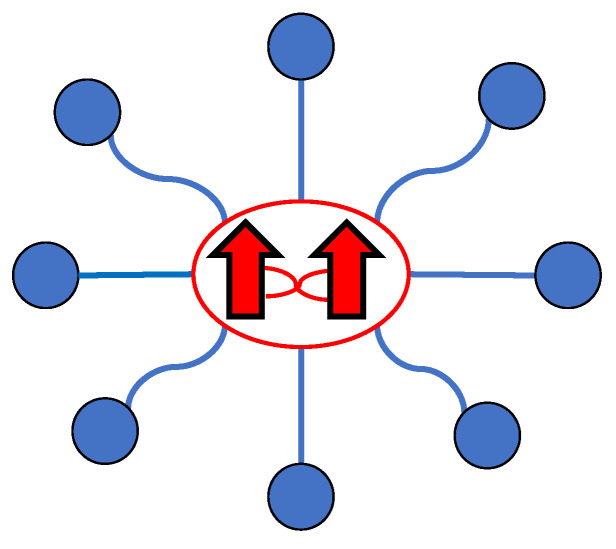
Schematic representation of the two-qubit spin-boson model. The two central arrows represent the two interacting qubits. The quantum harmonic oscillators constituting the bath are represented by the blue circles.

Let us consider the following open XYZ Heisenberg quantum model (in units of *ℏ*)

(1) $$\begin{array}{cc} H=\; & \Omega_{1}{\hat{\sigma }}_{1}^{z}+\Omega_{2}{\hat{\sigma }}_{2}^{z}+\sum_{j=1}^{N}\omega_{j}\;{\hat{a}}_{j}^{†}{\hat{a}}_{j}+\\ \; & \gamma_{x}{\hat{\sigma }}_{1}^{x}{\hat{\sigma }}_{2}^{x}+\gamma_{y}{\hat{\sigma }}_{1}^{y}{\hat{\sigma }}_{2}^{y}+\gamma_{z}{\hat{\sigma }}_{1}^{z}{\hat{\sigma }}_{2}^{z}+\sum_{k=1}^{2}\sum_{j=1}^{N}c_{ij}\left({\hat{a}}_{j}^{†}+{\hat{a}}_{j}\right){\hat{\sigma }}_{i}^{z}, \end{array}$$

describing two interacting spin-1/2’s subjected to local longitudinal (along the *z* direction) fields and coupled to a common reservoir. A pictorial representation of the system is shown in Figure 1.

${\hat{\sigma }}_{k}^{x}$, ${\hat{\sigma }}_{k}^{y}$ and ${\hat{\sigma }}_{k}^{z}$ (k=1,2) are the Pauli matrices, and $a_{j}$ and $a_{j}^{†}$ are the annihilation and creation boson operators of the *j*-th field mode. $\Omega_{i}$ and $\omega_{j}$ are the characteristic frequencies of the two spin-qubits (i=1,2) and the *j*-th field mode, respectively, and $\gamma_{k}$ (k=x,y,z) are the three real parameters characterizing the spin–spin anisotropic Heisenberg interaction.

The Hamiltonian is invariant when each spin is rotated by π around the $\hat{z}$-axis. The unitary operator accomplishing such a transformation is [41,42,43,44,45]

(2) $$e^{i\pi {\hat{\sigma }}_{1}^{z}/2}⊗e^{i\pi {\hat{\sigma }}_{2}^{z}/2}=-{\hat{\sigma }}_{1}^{z}{\hat{\sigma }}_{2}^{z}=\cos\left(\frac{\pi }{2}{\hat{\sum }}_{z}\right),$$

where ${\hat{\sum }}_{z}\equiv {\hat{\sigma }}_{1}^{z}+{\hat{\sigma }}_{2}^{z}$. Thus ${\hat{\sigma }}_{1}^{z}{\hat{\sigma }}_{2}^{z}$ is a constant of motion, which in turn implies the existence of two dynamically invariant subspaces ($H_{a}$ and $H_{b}$) related to its two eigenvalues (±1). It is possible to see that the two effective Hamiltonians, governing the dynamics of the two-qubit/bath system in each subspace, read [41]

(3) $$\begin{array}{c} H_{a}=\Omega_{a}{\hat{\sigma }}_{a}^{z}+\gamma_{a}{\hat{\sigma }}_{a}^{x}+\gamma_{z}{\hat{1}}_{a}+\sum_{j=1}^{N}\omega_{j}\;{\hat{a}}_{j}^{†}{\hat{a}}_{j}+\sum_{j=1}^{N}c_{j}^{a}\left({\hat{a}}_{j}^{†}+{\hat{a}}_{j}\right){\hat{\sigma }}_{a}^{z}, \end{array}$$

for $\sigma_{1}^{z}\sigma_{2}^{z}=1$, and

(4) $$\begin{array}{c} H_{b}=\Omega_{b}{\hat{\sigma }}_{b}^{z}+\gamma_{b}{\hat{\sigma }}_{b}^{x}+\gamma_{z}{\hat{1}}_{b}+\sum_{j=1}^{N}\omega_{j}\;{\hat{a}}_{j}^{†}{\hat{a}}_{j}+\sum_{j=1}^{N}c_{j}^{b}\left({\hat{a}}_{j}^{†}+{\hat{a}}_{j}\right){\hat{\sigma }}_{b}^{z}, \end{array}$$

for $\sigma_{1}^{z}\sigma_{2}^{z}=-1$, with

(5) $$\begin{array}{cc} \Omega_{a/b}=\Omega_{1}\pm \Omega_{2}, & \;\gamma_{a/b}=\gamma_{x}\pm \gamma_{y},\;c_{j}^{a/b}=c_{1j}\pm c_{2j}. \end{array}$$

The original Hamiltonian can be then written as $H=H_{a}⊕H_{b}$. Thus, the dynamics of the two-spin/bath system in each subspace can be simulated by that of an effective single-impurity spin-boson model (SISBM), that is a fictitious two-level system immersed in a fictitious magnetic field and coupled with a reservoir through effective coupling constants. In this case, the role of the effective transverse field is played by the coupling existing between the two spin-1/2’s. It is important to underline that, although the two effective Hamiltonians are qualitatively similar, they can deeply differ under specific physical conditions, leading to a remarkably different dynamics of the physical system described by Equation (1) in the two different subspaces of the two-spin/bath system.

The subspace *a* related to the eigenvalue +1 of ${\hat{\sigma }}_{1}^{z}{\hat{\sigma }}_{2}^{z}$ is spanned by

(6) $$\begin{array}{c} \{|++\rangle ,|--\rangle \}{⊗}_{j=1}^{N}\{|n_{j}\rangle {\}}_{n=0}^{\infty }, \end{array}$$

with ${\hat{\sigma }}^{z}|\pm \rangle =\pm |\pm \rangle$, and ${\hat{a_{j}}}^{†}\hat{a_{j}}|n_{j}\rangle =n_{j}|n_{j}\rangle$ (j=1,…,N). It means that the two states {|++〉,|−−〉} are mapped into the states ${\{|+\rangle }_{a}{,|-\rangle }_{a}\}$ of the fictitious two-level system *a*. So, in this case, by studying the dynamics of the fictitious spin-1/2 *a* effectively coupled to a bath, we study the dynamics of the two-spin/bath system within the subspace $H_{a}$ ruled by the effective Hamiltonian (3). Analogously, the subspace $H_{b}$ related to the eigenvalue −1 of ${\hat{\sigma }}_{1}^{z}{\hat{\sigma }}_{2}^{z}$ is spanned by the two-spin/bath states

(7) $$\begin{array}{c} \{|+-\rangle ,|-+\rangle \}{⊗}_{j=1}^{N}\{|n_{j}\rangle {\}}_{n=0}^{\infty }, \end{array}$$

and the effective Hamiltonian ruling the dynamics is that given in Equation (4). In this case, the two states ${\{|+\rangle }_{b}{,|-\rangle }_{b}\}$ of the fictitious spin-1/2 *b* are the mapping images of the two two-spin states {|+−〉,|−+〉}, respectively. Thanks to such a dynamic separation, the time evolution from initial conditions involving the two invariant subspaces can be easily achieved.

Finally, it is worth pointing out that, being each subdynamics ruled by an effective spin-boson Hamiltonian, all the results obtained for the spin-boson model can be applied to each subdynamics and interpreted in terms of the two interacting spin-1/2’s. Then, we can disclose the dynamics of the two-spin/bath system by separately solving the two effective spin-boson dynamical problems and ‘merging’ the obtained results.

## 3. Exactly Solvable Case

In this section we specialize the model (1) making it invariant under the two spin exchange. The simplest way to reach this goal is to introduce the following physically transparent links between the parameters appearing in *H*:

(8) $$\Omega_{1}=\Omega_{2}=\Omega /2,\;\gamma_{x}=\gamma_{y}=\gamma /2,\;c_{1j}=c_{2j}=c_{j}/2\;\left(\forall j\right).$$

For this exchange-symmetry case the Hamiltonian of the two-spin/bath system reads

(9) $$\begin{array}{cc} H= & \frac{\Omega }{2}\left({\hat{\sigma }}_{1}^{z}+{\hat{\sigma }}_{2}^{z}\right)+\sum_{j=1}^{N}\omega_{j}\;{\hat{a}}_{j}^{†}{\hat{a}}_{j}+\\ \; & \frac{\gamma }{2}\left({\hat{\sigma }}_{1}^{x}{\hat{\sigma }}_{2}^{x}+{\hat{\sigma }}_{1}^{y}{\hat{\sigma }}_{2}^{y}\right)+\gamma_{z}{\hat{\sigma }}_{1}^{z}{\hat{\sigma }}_{2}^{z}+\sum_{j=1}^{N}\frac{c_{j}}{2}\left({\hat{a}}_{j}^{†}+{\hat{a}}_{j}\right)\left({\hat{\sigma }}_{1}^{z}+{\hat{\sigma }}_{2}^{z}\right). \end{array}$$

With respect to Hamiltonian (1), where only the square of ${\hat{\sum }}_{z}$ is a constant of motion, Hamiltonian (9) exhibits a higher symmetry, being also the *z*-component of the total spin a constant of motion. The two effective Hamiltonians, which now rule the dynamics of the two-spin/bath system in the two invariant subspaces (still definable), become

(10) $$\begin{array}{cc} H_{a} & =\Omega {\hat{\sigma }}_{a}^{z}+\gamma_{z}{\hat{1}}_{a}+\sum_{j=1}^{N}\omega_{j}\;{\hat{a}}_{j}^{†}{\hat{a}}_{j}+\sum_{j=1}^{N}c_{j}\left({\hat{a}}_{j}^{†}+{\hat{a}}_{j}\right){\hat{\sigma }}_{a}^{z},\\ H_{b} & =\gamma {\hat{\sigma }}_{b}^{x}-\gamma_{z}{\hat{1}}_{b}+\sum_{j=1}^{N}\omega_{j}\;{\hat{a}}_{j}^{†}{\hat{a}}_{j}. \end{array}$$

We underline that, under the particular physical conditions in Equation (8), $H_{b}$ presents an effective decoupling of the fictitious two-level system *b* (which simulates the behaviour of the two actual spins within the subspace *b*) from the relative bosonic bath. This means that the subspace *b* is a decoherence-free subspace, or, in other words, that any initial state of the two-spin system belonging to such a subspace evolves as if the coupling between the two spins and the bath were absent [46,47,48]. The physical reason at the basis of this occurrence relies on the equal coupling of the two spins to the bath, i.e., $c_{1j}=c_{2j},\;\forall j$. The two couplings in a certain sense compensate each other, canceling out the effective spin-bath interaction in the subspace $H_{b}$.

Moreover, we note that both Hamiltonians in Equation (10) result to be exactly diagonalizable. The homogeneity of the longitudinal magnetic field applied to the spin pair, namely $\Omega_{1}=\Omega_{2}$, causes ${\hat{\sigma }}_{a}^{z}$ to be a constant of motion of $H_{a}$. Thanks to the isotropy of the spin–spin coupling strength, i.e., $\gamma_{x}=\gamma_{y}$, ${\hat{\sigma }}_{b}^{x}$ is instead a constant of motion of $H_{b}$. The subspace $H_{a}$ ($H_{b}$) can be then separated in two invariant subspaces, $H_{a}^{\pm }$ ($H_{b}^{\pm }$), labeled by the two eigenvalues (±1) of the corresponding constant of motion ${\hat{\sigma }}_{a}^{z}$ (${\hat{\sigma }}_{b}^{x}$). Therefore, the total Hilbert space H is the direct sum of these four infinite-dimensional, dynamically invariant Hilbert subspaces, namely

(11) $$H=H_{a}^{+}⊕H_{a}^{-}⊕H_{b}^{+}⊕H_{b}^{-}.$$

We underline that these four orthogonal invariant subspaces are identified and characterized by constant values of physical variables related only to the two-spin system. A consequence of Equation (11) is that, preparing the two-spin/bath system in a factorized spin-bath state belonging to one out of the four invariant subspaces given in Equation (11), the evolved state is still in a factorized form and the two-spin factor is stationary.

The two Hamiltonians

(12) $$\begin{array}{cc} H_{a}^{\pm } & =\pm \Omega +\gamma_{z}{\hat{1}}_{a}+\sum_{j=1}^{N}\omega_{j}\;{\hat{a}}_{j}^{†}{\hat{a}}_{j}\pm \sum_{j=1}^{N}c_{j}\left({\hat{a}}_{j}^{†}+{\hat{a}}_{j}\right), \end{array}$$

corresponding to the two eigenvalues of ${\hat{\sigma }}_{a}^{z}$, must be intended as the effective Hamiltonians governing the dynamics of the two-spin/bath system within the subspace $H_{a}^{+}$ and $H_{a}^{-}$, respectively. The eigenstates (written in terms of the two-qubit/bath states, in view of the mapping discussed in Section 2) and eigenenergies of these two Hamiltonians can be written, respectively, as

(13) $$\begin{array}{cc} |\psi_{\left\{n_{j}\right\}}^{a\pm }\rangle & =|\pm \pm \rangle {⊗}_{j=1}^{N}D\left(\mp \alpha_{j}\right)\frac{{\left({\hat{a}}_{j}^{†}\right)}^{n_{j}}}{\sqrt{n_{j}!}}|0_{j}\rangle ,\\ E_{\left\{n_{j}\right\}}^{a\pm } & =\pm \Omega +\gamma_{z}+\sum_{j=1}^{N}\left(n_{j}-\alpha_{j}^{2}\right)\omega_{j} \end{array}$$

with $D(\alpha_{j})$ ($\alpha_{j}=c_{j}/\omega_{j}$) and $|0_{j}\rangle$ being the displacement operator and the vacuum state of the *j*-th quantized bosonic mode, respectively [49]. Considering an infinite number of harmonic oscillators of the bath (N→∞), the last term in the expression of the eigenenergies diverges. In order to obtain a finite esteem of this energy contribution, we consider an Ohmic spectral density, that is $J\left(\omega \right)=\sum_{j}c_{j}^{2}\delta \left(\omega -\omega_{j}\right)\equiv \alpha \omega e^{\omega /\omega_{c}}$ (α and $\omega_{c}$ are the dimensionless parameter accounting for the spin-bath coupling strength and the cut-off energy of the bath, respectively). With this choice the energy contribution under scrutiny is finite and can be exactly derived, namely $\sum_{j}\alpha_{j}^{2}\omega_{j}\to \alpha \omega_{c}$ (the sum is replaced by the integral, namely $\sum_{j}\to \int J\left(\omega \right)d\omega$ [50,51]). In this case the lowest-energy states and the corresponding energies of the two Hamiltonians are uniquely defined and read

(14) $$\begin{array}{cc} |\psi_{\left\{0_{j}\right\}}^{a\pm }\rangle & \equiv |\psi_{0}^{a\pm }\rangle =|\pm \pm \rangle {⊗}_{j=1}^{N}D\left(\mp \alpha_{j}\right)|0_{j}\rangle ,\\ E_{\left\{0_{j}\right\}}^{a\pm } & \equiv E_{0}^{a\pm }=\pm \Omega +\gamma_{z}-\alpha \omega_{c}. \end{array}$$

We underline that the assumption of the Ohmic spectral density has been done only to obtain a finite esteem of the energy contribution of the bath (composed of an infinite number of quantum harmonic oscillators, N→∞). However, this choice does not affect the exact solvability of our model. Indeed, if we had considered a finite number of harmonic oscillators the expressions of the eigenenergies would have been the same, and the assumption of the Ohmic spectral density would not has been necessary, since the energy contribution of the bath would result finite.

The eigenstates (mapped into the two-qubit/bath language) and the corresponding eigenenergies of the two Hamiltonians $H_{b}^{\pm }$ related to the two eigenvalues of ${\hat{\sigma }}_{b}^{x}$ read instead

(15) $$\begin{array}{cc} |\psi_{\left\{n_{j}\right\}}^{b\pm }\rangle & =\frac{|+-\rangle \pm |-+\rangle }{\sqrt{2}}{⊗}_{j=1}^{N}\frac{{\left({\hat{a}}_{j}^{†}\right)}^{n_{j}}}{\sqrt{n_{j}!}}|0_{j}\rangle ,\\ E_{\left\{n_{j}\right\}}^{b\pm } & =\pm \gamma -\gamma_{z}+\sum_{j}n_{j}\omega_{j}, \end{array}$$

with lowest-energy states and corresponding energies given by

(16) $$\begin{array}{cc} |\psi_{\left\{0_{j}\right\}}^{b\pm }\rangle & \equiv |\psi_{0}^{b\pm }\rangle =\frac{|+-\rangle \pm |-+\rangle }{\sqrt{2}}{⊗}_{j=1}^{N}|0_{j}\rangle ,\\ E_{\left\{0_{j}\right\}}^{b\pm } & \equiv E_{0}^{b\pm }=\pm \gamma -\gamma_{z}. \end{array}$$

Finally, it is worth noticing that, although the two effective Hamiltonians $H_{a}$ and $H_{b}$ simplify under the considered conditions (resulting to be analytically diagonalizable), the original two-spin/bath Hamiltonian does not acquire a trivial form, from which it is easy to understand the exact solvability of the model. Rather, it is the decomposition procedure and the effective description in terms of fictitious systems which allow to easily read and recognize simple structures, leading to the solution of the dynamical problem.

### Quantum Phase Transitions

Thanks to the analytical solutions we can investigate and exactly derive the occurrence of quantum phase transitions. We consider the case of full isotropy in the spin–spin coupling, that is assuming $\gamma_{z}=\gamma_{x}=\gamma_{y}=\gamma /2$ in the Hamiltonian (9). In this instance the four lowest energies within each invariant subspace read

(17) $$E_{0}^{a+}=\Omega +\gamma /2-\alpha \omega_{c},\;E_{0}^{a-}=-\Omega +\gamma /2-\alpha \omega_{c},\;E_{0}^{b+}=\gamma /2,\;E_{0}^{b-}=-3\gamma /2.$$

**Figure 2:**
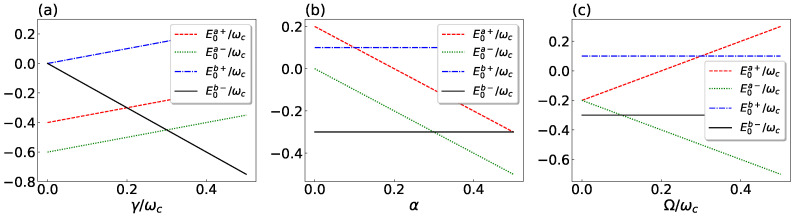
Dependence of the eigenenergies $E_{0}^{a+}$ (dashed red line), $E_{0}^{a-}$ (dotted green line), $E_{0}^{b+}$ (dot-dashed blue line), and $E_{0}^{b-}$ (solid black line) on the dimensionless (**a**) spin–spin coupling $\gamma /\omega_{c}$, (**b**) spin–bath coupling α, and (**c**) the (classical) magnetic field strength $\Omega /\omega_{c}$.

In Figure 2a–c the dependence of the above four eigenenergies is plotted as a function of the spin–spin coupling γ, the spin-bath coupling α, and the externally applied magnetic field Ω, respectively. It is possible to see that several level crossings occur. In particular, the one appearing between the two eigenenergies $E_{0}^{a-}$ (dotted green line) and $E_{0}^{b-}$ (solid black line) is of remarkable importance since it involves the ground state of the two-spin/bath system, giving rise to a first-order QPT.

Specifically, in Figure 2a we see that $E_{0}^{a-}<E_{0}^{b-}$ for $\gamma <\gamma_{c}$, while $E_{0}^{a-}>E_{0}^{b-}$ for $\gamma >\gamma_{c}$ ($\gamma_{c}$ being the crossing point, that is, the critical value of γ for which the ground state is degenarate and then not uniquely defined). Therefore, the ground state corresponds to $|\psi_{0}^{a-}\rangle$ for $\gamma <\gamma_{c}$ and to $|\psi_{0}^{b-}\rangle$ for $\gamma >\gamma_{c}$. It means that, in this case, the ground state ‘moves’ from $H_{a}^{-}$ to $H_{b}^{-}$ by increasing the spin–spin coupling γ and crossing the critical point corresponding to the critical value $\gamma_{c}$. This fact suggests that the ground state can belong to different invariant subspaces with respect to the parameter-space region we consider.

On the other side, by increasing the spin-bath coupling α, the opposite change occurs: $E_{0}^{a-}>E_{0}^{b-}$ and $E_{0}^{a-}<E_{0}^{b-}$ before and after, respectively, the crossing point $\alpha_{c}$ (see Figure 2b). The same transition is observed by varying the parameter Ω around its critical values $\Omega_{c}$ (see Figure 2c). The ground state is thus placed in $H_{b}^{-}$ (being $|\psi_{0}^{b-}\rangle$) for $\alpha <\alpha_{c}$ and $\Omega <\Omega_{c}$. Then, it ‘moves’ to $H_{a}^{-}$ (becoming $|\psi_{0}^{a-}\rangle$), after crossing the critical values of the two parameters, that is for $\alpha >\alpha_{c}$ and $\Omega >\Omega_{c}$ (see Figure 2b and Figure 2c, respectively).

These crossings are of course accompanied by relevant physical changes in the ground state properties of the system. The two states $|\psi_{0}^{a-}\rangle$ and $|\psi_{0}^{b-}\rangle$ are indeed characterized by different values of three relevant physical observables, namely: (i) the level of entanglement between the two spins estimated through the concurrence $C_{0}^{x-}$ [40] which, in case of a general two-qubit state

(18) $$|\psi \rangle =c_{++}|++\rangle +c_{+-}|+-\rangle +c_{-+}|-+\rangle +c_{--}|--\rangle ,$$

with $|c_{++}{|}^{2}+|c_{+-}{|}^{2}+|c_{-+}{|}^{2}+{\left|c_{--}\right|}^{2}=1$, reads

(19) $$C=2|c_{++}c_{--}-c_{+-}c_{-+}|;$$

(ii) the net two-spin magnetization $M_{0}^{x-}\equiv \langle \psi_{0}^{x-}\left|{\hat{\sum }}^{z}\right|\psi_{0}^{x-}\rangle$; (iii) the mean number of photons (or excitations) $N_{0}^{x-}\equiv \langle \psi_{0}^{x-}\left|{\hat{a}}^{†}\hat{a}\right|\psi_{0}^{x-}\rangle$ of the boson field (x=a,b).

By explicitly calculating on $|\psi_{0}^{a-}\rangle$ the average values of the three physical observable listed above, we obtain:

(20) $$\{C_{0}^{a-}=0,\;M_{0}^{a-}=-1,\;N_{0}^{a-}=\sum_{j=1}^{N}\alpha_{j}^{2}\}.$$

The same calculation performed for the state $|\psi_{0}^{b-}\rangle$ gives

(21) $$\{C_{0}^{b-}=1,\;M_{0}^{b-}=0,\;N_{0}^{b-}=0\}.$$

We see that the three physical quantities considered show different values for the two states which alternately result to be the ground state of the two-spin/bath system, depending on the parameter-space region taken into account. These observables then undergo an abrupt change in correspondence of the level-crossings occurring at the critical values of the control parameters. Therefore, we can speak of two different phases and then of first-order QPTs (at the crossing points) characterized by: (i) γ, α, and Ω as control parameters; (ii) the three physical quantities $C_{0}$, $M_{0}$, and $N_{0}$ as order parameters; (iii) the two states $|\psi_{0}^{a-}\rangle$ and $|\psi_{0}^{b-}\rangle$ as ground states of the two-spin/bath system in the two different phases.

We point out that the thermodynamic limit is not necessary, in general, for first-order QPTs [11]. In Refs. [52,53,54,55,56] the authors, studying the quantum Rabi model, make clear the difference between first-order QPTs occurring in the thermodynamic (classical) limit (vanishing frequencies for the bosonic field) and the first-order QPTs occurring in the fully quantum regime (that is, at finite frequencies). Precisely, they bring to light how the phase diagram (and then the physical feature) of the ground state of the system is substantially and remarkably different in the two regimes [52,53,54,55,56]. This circumstance clearly shows how few-body first-order QPTs offer a rich field of study to understand both semiclassical and fully quantum physical characteristics of microscopic light-matter systems.

In our case, however, the system is constituted by two interacting qubits and a multi-mode bosonic field. We underline that the bath is not a reservoir playing a passive role in the dynamics of the system. Its state is indeed not fixed and constantly equal to a thermal state, as in the case of a reservoir. We have indeed different states for the bath in the two ground states of the system related to the two different phases. Further, since the bath consists of an infinite number of quantum harmonic oscillators, the size of the system is then not finite. To esteem the energy contribution of the bath we have indeed taken the limit N→∞, after assuming an Ohmic spectral density. In this case thus the QPTs brought to light can be intended occurring in the thermodynamic limit.

It is particularly worth pointing out the different level of entanglement between the spins, which characterizes the two phases: the two spins are in a disentangled state in the space $H_{a}^{-}$, while exhibit a maximally entangled state in the space $H_{b}^{-}$. Furthermore, the (abrupt) change from a vanishing to a non-vanishing value (or vice versa) of the mean photon number suggests the occurrence of a superradiant QPT. Precisely, the phase corresponding to the ground state $|\psi_{0}^{b-}\rangle$ can be intended as the normal phase, while that corresponding to the ground state $|\psi_{0}^{a-}\rangle$ can be thought of as the superradiant phase [57]. The peculiarity of such a superradiant QPT lies in the fact that it is a first-order QPT [58,59], differently from both the standard superradiant phase transition of the Dicke model [60,61,62] and the superradiant QPT of the quantum Rabi model [57], which are instead phase transitions of the second order.

## 4. Conclusions

The present work studies a model which describes an ubiquitous physical situation that can be formalized in terms of two interacting qubits coupled to a common bosonic field. We have shown that, despite its non-triviality, the model turns out to be exactly solvable when a full symmetry between the two spins is considered. Precisely, we have focused on an isotropic Heisenberg spin–spin interaction, an homogeneous magnetic field applied to the spin pair, and an equal coupling of the spin–boson type between each spin and each mode of the field. In this instance it is then possible to analytically derive eigenenergies and eigenstates of the system. Thanks to our exact approach it has been possible to unveil the occurrence of QPTs with respect to the three parameters characterizing the model (the spin–spin coupling, the spin-bath coupling, and the strength of the classical magnetic field). These QPTs are characterized by abrupt changes in relevant physical observables of the system: the two-spin concurrence, the net spin magnetization, and the mean photon number. Such sudden changes are due to the fact that these QPTs are of the first order (presence of a level crossing). We have in particular emphasized that the change of the mean photon number from a vanishing to a non-vanishing value between the two phases allows to speak of superradiant phase transition. The peculiarity of such a superradiant phenomenon is related to the fact that it corresponds to a first-order QPT, differently from what happens for both the standard superradiant phase transition of the Dicke model [60,61,62] and the superradiant QPT of the quantum Rabi model [57], which are instead second-order phase transitions.

We underline that the analytical approach [63,64,65,66,67] used here allowed to exactly solve the time-independent Shrödinger equation. The method is further valid in every region of the parameter space: no constraints about the strength of the Hamiltonian parameters have been indeed introduced. The only requirement is the totally symmetric role of the two spins.

Since not so many exactly solvable (non-trivial) models, treating the coupling with a bath, are present in literature, our study can be then at the basis of an interesting insight on physics of open quantum systems. Moreover, thanks to its generality, our model can be applied to a plethora of physical systems useful for future quantum technologies. Until now, the two-spin-boson models considered in literature [17,27,32] have taken into account either decoupled qubits or the simplest spin–spin coupling. In our case, instead, the physical effects stemming from the presence of a (non-trivial) isotropic dipolar spin–spin coupling has been investigated for the first time. This aspect is of crucial importance since in some contexts the spin–spin interaction cannot be neglected, and in other scenarios, such as in quantum computation, it is fundamental to perform two-qubit quantum logic gates [35,36] and to generate entangled states of the system [37,38,39].

Finally, our exactly solvable model can be also exploited to test the accuracy of the standard techniques employed for mathematically treating open quantum systems, such as: (i) the standard Lindblad theory [68,69]; (ii) the partial Wigner transform [70,71,72]; (iii) the non-Hermitian formalism [73,74,75,76,77,78,79,80,81]; (iv) the stochastic approach [82,83,84,85,86,87,88].

## Data Availability

Not applicable.
